# Network pharmacology and in vitro experimental verification unveil glycyrrhizin from glycyrrhiza glabra alleviates acute pancreatitis via modulation of MAPK and STAT3 signaling pathways

**DOI:** 10.1186/s12906-024-04372-x

**Published:** 2024-01-27

**Authors:** Rui Zhang, Aiminuer Asikaer, Qi Chen, Fang Wang, Junjie Lan, Yang Liu, Linfang Hu, Huaye Zhao, Hongtao Duan

**Affiliations:** 1 Department of pharmacy, Guizhou Provincial People’s Hospital, Guiyang, 550002 China; 2 School of Pharmacy and Bioengineering, Chongqing University of Technology, Chongqing, 405400 PR China; 3 College of Stomotology, Zunyi Medical University, Zunyi, 563000 China; 4 Department of Hepatobiliary Surgery II, Guizhou Provincial People’s Hospital, Guiyang, 550002 China

**Keywords:** Acute pancreatitis, Glycyrrhizin, Network pharmacology, Molecular dynamics, Mitogen-activated protein kinase 3

## Abstract

Acute pancreatitis (AP) is a severe gastrointestinal inflammatory disease with increasing mortality and morbidity. Glycyrrhiza glabra, commonly known as Liquorice, is a widely used plant containing bioactive compounds like Glycyrrhizin, which possesses diverse medicinal properties such as anti-inflammatory, antioxidant, antiviral, and anticancer activities. The objective of this study is to investigate the active components, relevant targets, and underlying mechanisms of the traditional Chinese medicine Glycyrrhiza glabra in the treatment of AP. Utilizing various computational biology methods, we explored the potential targets and molecular mechanisms through Glycyrrhizin supplementation. Computational results indicated that Glycyrrhizin shows promising pharmacological potential, particularly with mitogen-activated protein kinase 3 (MAPK3) protein (degree: 70), forming stable complexes with Glycyrrhizin through ionic and hydrogen bonding interactions, with a binding free energy (ΔG_bind_) of -33.01 ± 0.08 kcal/mol. Through in vitro experiments, we validated that Glycyrrhizin improves primary pancreatic acinar cell injury by inhibiting the MAPK/STAT3/AKT signaling pathway. Overall, MAPK3 emerges as a reliable target for Glycyrrhizin’s therapeutic effects in AP treatment. This study provides novel insights into the active components and potential targets and molecular mechanisms of natural products.

**Supplementary Information:**

The online version contains supplementary material available at 10.1186/s12906-024-04372-x.

## Introduction

Acute pancreatitis (AP) is a potentially life-threatening condition characterized by local injury and inflammation of the pancreas [1, 2]. Despite extensive research, the underlying mechanisms driving its development and progression remain unclear, and currently available treatments lack specificity and efficacy [3]. Key pathological features of AP include the inappropriate activation of zymogens within the pancreas, impaired and dysregulated secretion of digestive enzymes, accumulation of vacuoles, inflammation, and the occurrence of apoptotic and necrotic acinar cell death [4]. Given the absence of a licensed drug resulting from numerous clinical trials, there is a pressing need to search for potential bioactive compounds to effectively manage and mitigate the global impact of acute pancreatitis.

In China, the utilization of Traditional Chinese Medicine (TCM) is a widely recognized and established approach in the management of acute pancreatitis (AP). Dachengqi decoction [5], chaiqin chengqi decoction [6], qingyi decoction, and yinchenchengqi decoction have been successfully employed for over three decades in the treatment of AP patients [7, 8]. Within the framework of traditional Chinese medicine, Glycyrrhiza glabra holds a revered status as an indispensable herbal remedy due its remarkable capacity to mitigate toxicity and enhance the therapeutic effectiveness of co-administered herbal medicines [9]. Glycyrrhiza glabra, commonly referred to as Liquorice, is extensively employed in Mediterranean regions, India, Russia, and Central Asia, making it one of the most globally consumed herbal remedies [10, 11]. The extracts of Glycyrrhiza glabra have found applications in the food and pharmaceutical industries, and have been utilized in the development of nutritional supplements and functional foods. The historical utilization of liquorice in traditional medicine and folk remedies has left a lasting impact [12]. Within traditional Chinese medicine, it is commonly recommended for addressing gastrointestinal ailments, coughs, bronchitis, and arthritis. Moreover, recent clinical research has highlighted potential benefits of liquorice extracts, including the improvement of body balance control [13], anti-asthmatic properties [14], anti-influenza activity [15], enhanced innate immunity in COVID-19 patients [16, 17], and promotion of pulmonary functions [18].

Liquorice exhibits noteworthy anti-inflammatory effects attributed to its ability to reduce pro-inflammatory factors and free radicals. An ethanol extract derived from liquorice demonstrated improved survival rates in LPS-treated mice, accompanied by reduced plasma levels of TNF-α and IL-6, as well as increased production of IL-10 [19]. Furthermore, the liquorice extract has demonstrated the ability to inhibit the phosphorylation of macrophages involved in intracellular signaling pathways of inflammatory proteins, including NF-κB p65 nuclear and Jun proto-oncogene-encoded activator protein (AP-1) transcription factor [20]. Notably, bioactives of liquorice were reported to exhibited anti-pancreatitis activities. Glycyrrhizin, a main active ingredient of liquorice, was shown to suppress the expressions of HMGB1 and alleviate the severity of traumatic pancreatitis in rats [21, 22]. Another bioactive isoliquiritigenin was demonstrated to ameliorates acute [23] pancreatitis by inhibition of oxidative stress, it also relief chronic pancreatitis [24] by preventing pancreatic stellate cells activation and macrophages infiltration. These studies support the idea of utilizing of liquorice or extracts of liquorice for the treatment of acute pancreatitis.

Although there is considerable interest in exploring the efficacy of the active constituents found in liquorice for the treatment of acute pancreatitis, a significant portion of the research predominantly revolves around reinterpreting existing knowledge. Considering the extensive range of potential implications and mechanisms, exclusively using experimental screening techniques proves to be both resource-intensive and time-consuming. Moreover, liquorice’s diverse effects are attributed to multiple ingredients, posing challenges in the identification of bioactive substances and their underlying mechanisms. Consequently, there is a pressing need to systemically explore the medicinal impacts and elucidate the key mechanisms of liquorice for the treatment of acute pancreatitis. Network pharmacology, an interdisciplinary approach that integrates systems biology methods, has sprang up as a valuable instrument for comprehending the health benefits of drugs within complex biological systems. Specifically, it has found extensive application in the investigation and disclosure of remedy effects and mechanisms associated with Herb-based therapies [25], facilitating a wide range of investigations such as the characterization of active compounds and prediction of target candidates [26]. Molecular docking is a computational technique used to predict the optimal binding orientation and affinity between ligand molecules and receptor proteins. It combines principles such as energy matching and geometric matching to assess the binding capacity between these entities [27]. On the other hand, molecular dynamics simulations are employed to study protein folding, structural stability, and interactions between proteins and small molecules, enabling a deeper understanding of the dynamic behavior of drug molecules in the biological context and revealing their functionalities and mechanisms [28]. In conclusion, the integration of diverse computational biology methods can synergistically enhance research in traditional Chinese medicine, offering innovative perspectives into the screening of active compounds and exploration of their modes of action.

In the present study, we utilized a comprehensive approach, including computational biology methods and in vitro experiments, to elucidate the primary bioactive constituents of Glycyrrhiza glabra for the treatment of AP and to uncover the underlying mechanisms. Our results indicate that MAPK3 emerges as a promising target for Glycyrrhizin in AP therapy, and maintaining the expression of MAPK3 in acinar cells to ameliorate cell necrosis could represent a novel and potential therapeutic strategy for AP treatment.

## Methods

### Animals

All the male, normal C57BL/6 mice (8–10 week old; 20–24 g body weight) used in the current study were obtained from Hunan SJA Experimental Animal Co., Ltd. (Chongqing, China). All animals were housed in cages under controlled conditions of temperature (23–25 °C) and humidity (50–60%), with a standard light/dark cycle of 12 h each. plenty of water and food were available in the cages throughout the study period. In the experimental process, a total of 10 mice were utilized, with five mice housed in each cage. All mice were subjected to the extraction of primary acinar cells from the pancreas.

### Primary pancreatic acinar cell isolation and culturing

To isolate pancreatic acinar cells from C57BL/6 mice, we employed a specific collagenase digestion procedure as previously described [29]. During the euthanasia procedure, mice were initially placed in a sealed chamber, and carbon dioxide (CO_2_) gas was introduced into the chamber. The concentration of CO_2_ rapidly reached 40%, inducing quick anesthesia in the mice. Subsequently, cervical dislocation was performed manually, resulting in spinal cord injury, ensuring the mice lost consciousness immediately and ceased respiration. Subsequently, the pancreas was meticulously dissected and rinsed thrice with phosphate-buffered saline (PBS). Subsequently, collagenase IV (200 U/mL) was gently infused into the pancreas via a sterile 1 mL syringe, directed along the duct to induce its expansion. The tissue was then subjected to a 37 °C water bath for a 19-minute digestion period. Following digestion, the pancreatic tissue was immersed in an extracellular solution with a composition of 140 mM NaCl, 4.7 mM KCl, 1.13 mM MgCl_2_, 1 mM CaCl_2_, 10 mM D-glucose, and 10 mM HEPES, maintaining a pH of 7.30. The digested pancreas was subjected to repetitive pipetting in this solution. The resultant suspension underwent sterile filtration through a cell strainer, employing a mechanical dissociation method to isolate cells. Subsequent to a mild centrifugation at 700 rpm for 2 min, primary acinar cells were obtained from the pancreas [30].

### Screening strategy for bioactive compounds and potential targets in glycyrrhiza glabra

The ingredients of Glycyrrhiza glabra were retrieved from a literature [31]. We screened the corresponding targets in TCMSP database and Swiss target prediction (http://www.swisstargetprediction.ch), and finally removed the duplicate values from the results of both databases to obtain the targets of active ingredients in Glycyrrhiza glabra [32].

### Target protein collection for AP and construction of protein-protein interaction (PPI) network

The targets associated with acute pancreatitis were collected from Drugbank (https://www.drugbank.ca), GeneCards (https://www.genecards.org), and OMIM (https://www.omim.org) databases separately. Duplicate values among the targets from the three databases were removed to identify potential targets for acute pancreatitis. All protein targets were then normalized using Uniprot (https://www.uniprot.org) and NCBI (https://www.ncbi.nlm.nih.gov) databases. The intersection of the normalized targets was analyzed using the String database (https://cn.string-db.org/), with the species set to *Homo sapiens*. The resulting data was visualized in Cytoscape 3.7.1 to identify significant targets based on higher Degree values [33].

### GO and KEGG enrichment analysis

The Gene Ontology (GO) and Kyoto Encyclopedia of Genes and Genomes (KEGG) pathway enrichment analyses were conducted using the DAVID database (https://david.ncifcrf.gov/). The DAVID database is a widely-used bioinformatics tool that provides comprehensive functional annotation and enrichment analysis of gene lists. It offers valuable insights into the biological processes (BP), cellular components (CC), and molecular functions (MF) associated with the submitted genes, as well as their involvement in important signaling pathways [34]. In this study, only data from the species “Homo sapiens” were included, and the enrichment of the pathway was considered significant when the modified Fisher exact false discovery rate (FDR) was less than 0.01. To analyze and visualize the data, an online bioinformatics platform (http://www.bioinformatics.com.cn/) was utilized [35].

### Construction of the glycyrrhiza glabra-compounds-targets-AP network

We imported the complex relationship between Glycyrrhiza glabra, compounds, targets and AP into Cytoscape 3.7.1 software (to construct a Glycyrrhiza glabra-Compounds-Targets-AP network), and the main components were identified according to the topological parameters [36].

### Molecular docking analysis

Molecular docking is a highly valuable technique in drug discovery, enabling accurate prediction of the conformation of small molecule ligands within the binding site of target proteins and assessment of their binding affinity [37]. In our study, we used AutoDock Vina to analyze the potential binding modes and key interactions between compounds and target proteins. The crystal structures of all target proteins were obtained from the Protein Data Bank (PDB, https://www.rcsb.org/). Subsequently, water molecules and ions were removed from the protein structures. Ligands were prepared using SYBYL-X 2.0 software and subjected to energy minimization. AutoDock tools were utilized to set rotatable bonds, merge non-polar hydrogen atoms, and add gas-phase charges for the components [38]. Subsequently, the target proteins and main components were converted into PDBQT format using AutoDock Tool software. Crystallographic docking grids were built for each target using AutoDock Tool. Finally, the docking results were visualized using PyMOL and Maestro 2021 academic version [39]. This semi-flexible docking approach considers the conformational changes of the ligand molecules, allowing exploration of their conformations during the calculations, thus enhancing the accuracy of the binding predictions.

### ADMET and density functional theory (DFT) calculations

The ADMET (Absorption, Distribution, Metabolism, Excretion and Toxicity) properties of drug molecules are crucial factors determining the success of drug development. In our research, we primarily utilize the online platform ADMET Lab 2.0 (https://admetmesh.scbdd.com) to evaluate the performance of compounds [40]. Human intestinal absorption (HIA) is a crucial parameter for assessing the in vivo absorption of drugs, and compounds with an absorption rate below 30% are generally regarded as poorly absorbed. Volume of distribution (VD) is an essential parameter describing the distribution of drugs in the body, with reference ranges between 0.04 and 20 L/kg. In terms of metabolism, as 80% of the isoenzymes belong to the human cytochrome P450 family, including 1A2, 3A4, 2C9, 2C19, and 2D6, the evaluation of compound metabolism in vivo can be determined by assessing their ability to be metabolized by these five P450 enzymes or serve as their inhibitors. Excretion is related to bioavailability and is mainly evaluated using clearance (CL) and half-life(T_*1/2*_) measures [41]. Additionally, toxicological assessments of the molecules can be conducted using AMES toxicity/skin sensitivity/hepatotoxicity indices. The optimal molecular structure of the ligand with the lowest energy was determined using the “FT (B3LYP with 6-31G(d)” basis set. The computational software Gaussian 03 W and GaussView 05 were employed to generate the most energetically favorable molecular structure [42]. Subsequently, in this study, the LUMO and HOMO energies, as well as the molecular electrostatic potential (MESP) representing the static electric characteristics of the ligand, were estimated [43]. The software programs Multiwfn 3.8 and VMD 1.9.3 were utilized for visualization and analysis of the obtained results.

### Molecular dynamics (MD) simulation

Currently, molecular dynamics (MD) simulations play a crucial role in the study of protein stability, protein folding, protein-ligand interactions, molecular recognition, and are increasingly pivotal in drug screening and discovery. MD simulations can significantly reduce the costs and enhance the efficiency of drug development [44]. For a given system, MD simulation involves determining the initial structure of the simulation, optimizing the starting structure, randomly assigning initial velocities to simulation objects, running an MD simulation for a specified time, calculating forces, using force calculations to compute accelerations, further combining initial velocities to calculate positions, and finally analyzing trajectories to draw conclusions. The total energy of the system is the sum of the molecular kinetic and potential energies for the N particles constituting the system. In the context of MD simulations, finite difference methods (such as Verlet algorithm, Leap-frog algorithm, correction-prediction algorithm, etc.) are essential for solving Newton’s equations of motion. This entire process entails generating various structures from a natural ensemble using a computed potential energy function. These generated structures are then employed for evaluation, with the assessment results representing the equilibrium of the system [45]. Molecular docking based on MD simulation can not only examine the process of ligand incorporation into protein, but also predict the process of ligand dissociation from protein, providing richer and more specific kinetic information for the study of the interaction between protein and ligand [46]. MD simulation is a powerful computational method that predicts the trajectories of particle coordinates, velocities, and energies over time, allowing for the analysis of molecular interactions and conformational changes [47]. The docking complex of glycyrrhizin in combination with the top affinity was further subjected to all-atom 100ns MD simulations with Amber22 [48].

Prior to the MD simulation, the compound underwent minimization using the HF/6-31G* optimization method in Gaussian 09, with resp fitting carried out using antechamber. Simultaneously, hydrogen atoms from all amino acid residues were eliminated, leaving only the coordinates of heavy atoms. The LEaP program in AmberTools was then employed to reconstruct the positions of hydrogen atoms. In order to enforce hydrogen bond constraints, we employed the SHAKE method. The total charge of the simulated system was neutralized by adding Na^+^ or Cl^−^ ions for charge balancing. The parameters of ligand generalized Amber force field 2 (GAFF2) are generated by Antechamber and tleap modules. The complex systems were placed in a TIP3PBox with a buffer greater than 10.0 Å, and the protein was parametrized using the ff19SB force field [49]. The system was then subjected to a series of energy minimization steps in the following order: solvent and ion minimization (4000 steps), solution and side chain minimization (5000 steps), and total system minimization (10,000 steps). These steps helped to relax the system and achieve a stable conformation for further molecular dynamics simulations [25]. The simulation system was rapidly heated to 300 K (50 ps). A combination of NVT is used to stabilize the density. The unrestrained production phase is then started, running 100 ns, i.e. One atmosphere and 303 K, with NPT ensemble [50]. The equilibrium in MD simulations was evaluated through various parameters, including the Root Mean Square Deviation (RMSD) of the receptor-ligand complex, Radius of Gyration (Rg), Solvent Accessible Surface Area (SASA), Root Mean Square Fluctuation (RMSF) of the receptor, important residue distances, and protein-ligand interactions. Additionally, Dynamic Cross-Correlation Matrix (DCCM) and Principal Component Analysis (PCA) of the trajectory were performed using the Bio 3D package in R to further assess the dynamics of the system during the simulation [51]. The Amber Tools software package was used, specifically the MMPBSA.py script, to analyze the binding free energy values of the simulation system. The MD sampling method was employed for free energy prediction using the MMPB/GBSA (Molecular Mechanics Poisson-Boltzmann/Generalized Born Surface Area) method [52]. This method is extensively employed and is particularly well-suited for precise calculations on large systems. Nonetheless, it does have some limitations and can be time-consuming due to the high computational cost associated with the system’s complexity [53]. The binding free energy equation for MMPB/GBSA is as follows:

$$\Delta {G_{bind}} = {G_{complex}} - {\text{ }}{G_{protein}} - {\text{ }}{G_{ligand}},$$

$$\Delta {G_{bind}} = \Delta {E_{MM}} + {\text{ }}\Delta {G_{GB}} + {\text{ }}\Delta {G_{SA}} - {\text{ }}T\Delta S$$

Where:

∆G_bind_ : Binding free energy of binding, G_complex_ : Free energies of the complex, G_protein_ : Free energy of the protein, G_ligand_ : Free energy of the ligand, ∆E_MM_ : Interaction energy between small molecules and proteins, including the electrostatic and van der Waals interaction energies, ∆G_GB_ : Difference between the polar solvation energy of the protein-ligand complex and the sum of the polar solvation energies of the protein and the ligand, ∆G_SA_ : Difference between the non-polar solvation free energy of the complex and the sum of the non-polar solvation free energies of the protein and the ligand, T∆S : Change in entropy of the ligand-bound conformation.

### Cell death assays by hoechst 33,342/PI staining

In this experiment, primary pancreatic acinar cells were exposed to sodium taurocholate (NaT) at a final concentration of 5 mM for 1 h, either alone or in combination with various concentrations of Glycyrrhizin. After loading the cells with Hoechst 33,342 (50 µg/mL) to stain the nuclei and propidium iodide (PI, 1 µM) to assess plasma membrane rupture, the cells were washed three times with PBS. The dye mixture was then discarded, and the cells were observed under a Fluorescence microscopy (Nikon, Tokyo, Japan) [54]. A minimum of 1 × 10^3^ cells were counted for each experimental condition to quantify the number of propidium iodide (PI)-positive cells in each field of view, thus assessing the extent of necrosis. Three independent replicates were performed for each condition, facilitating the calculation of the percentage of necrotic cells. This experiment utilized Image J software for the quantification of cells. The final calculation for cell death rate is expressed as follows:

$$\begin{gathered}Necrosis{\text{ }}Rate\left( \% \right) = \left( {PI - positive{\text{ }}cells{\text{ }}in{\text{ }}red} \right)/ \hfill \\\,\,\,\,\,\,\,\,\,\,\,\,\,\,\,\,\,\,\,\,\,\,\,\,\,\,\,\,\,\,\,\,\,\,\,\,\,\,\,\,\,\,\left( \begin{gathered}Total{\text{ }}Hoechst{\text{ }}33342 - \hfill \\stained{\text{ }}cells{\text{ }}in{\text{ }}blue \hfill \\ \end{gathered} \right) \times {\text{ }}100\% \hfill \\ \end{gathered}$$

### Western blot

To detect the change of expression of protein, the cells were challenged with lower concentration of NaT for longer time. After incubation with NaT (3 mM) for 12 h with or without glycyrrhizin, the acini were centrifuged, collected, and lysed with radioimmunoprecipitation assay buffer (RIPA). The total cell lysates (30 µg each) were loaded into individual wells of a 10-well comb. The protein expression levels of pancreatic acinar cells were assessed by measuring the levels of glyceraldehyde-3-phosphate dehydrogenase (GAPDH) (Proteintech Group, Chicago, IL). The samples were separated on 10% polyacrylamide SDS gels, and the proteins were then transferred to polyvinylidene fluoride (PVDF) membranes through electrophoretic transfer. Subsequently, the PVDF membranes were incubated in 5% skim milk at room temperature for 1 h with continuous shaking on a shaker during the entire blocking process. The membranes were then incubated overnight at 4 °C with specific primary antibodies. Afterward, appropriate horseradish peroxidase-conjugated secondary antibodies (Proteintech Group, Chicago, IL) were added and incubated for 1 h [55]. Subsequently, the signal images were detected using an enhanced chemiluminescence system (Amersham Image Quant 800, Cytiva). The primary antibodies GAPDH (Proteintech, 1:5000), ERK1/2, p-ERK1/2 (Vazyme, 1:2000), STAT3, p-STAT3 (Cell Signaling Technology, 1:1000), AKT, p-AKT (Cell Signaling Technology, 1:1000). Densitometric analysis was conducted using Image Lab Software (Version 1.4.2b, National Institutes of Health, USA) [55, 56].

### Statistical analysis

Quantitative data were presented as mean ± standard error of the mean (SEM). Statistical analyses were performed using GraphPad Prism 8.0.2 (GraphPad Software, USA) with one-way analysis of variance (ANOVA) followed by Dunnett’s least significant difference post hoc tests. A P-value < 0.05 was considered statistically significant. Randomization and blinding were employed whenever possible.

## Results

### Various active ingredients in glycyrrhiza glabra treat acute pancreatitis and involves multiple pathway pathways and targets

**Fig. 1:**
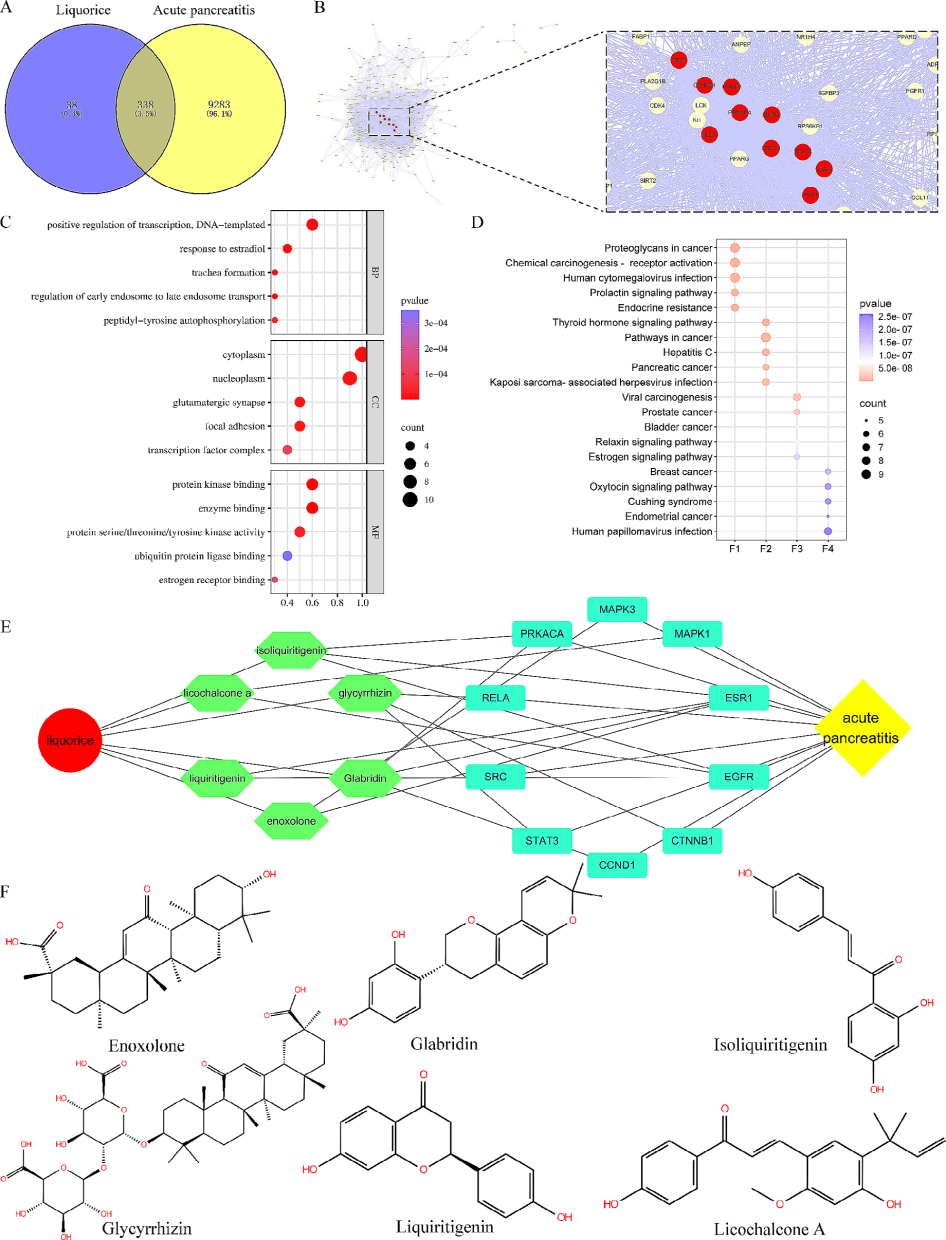
Interactions between various active ingredients in Glycyrrhiza glabra and acute pancreatitis involving multiple pathways and targets. **(A)** Venn diagram displaying the intersection of targets between Glycyrrhiza glabra (Liquorice) and acute pancreatitis. **(B)** Protein-protein interactions among the intersection targets, with the top 10 nodes highlighted in red. **(C)** GO enrichment analysis of the core targets, including Biological Process (BP), Cellular Component (CC), and Molecular Function (MF). **(D)** KEGG pathway enrichment analysis of the core targets. **(E)** Glycyrrhiza glabra (Liquorice) - active ingredients - target proteins - acute pancreatitis network. **(F)** Chemical structures of the active ingredients

Initially, we identified 338 intersecting targets by predicting the target proteins of all components of Glycyrrhiza glabra and those associated with acute pancreatitis in Fig. 1A. Subsequently, we performed protein-protein interaction (PPI) analysis on these 338 targets to explore their topological properties. The top ten targets based on degree centrality are shown in Fig. 1B; Table 1. Remarkably, we conducted GO and KEGG pathway enrichment analyses on these potential targets and found their involvement in diverse pathways. The top 5 enriched categories for BP, CC, and MF, as well as the top 20 KEGG pathways, are displayed in Fig. 1C-D using bubble plots. Particularly, significant pathways include “Proteoglycans in cancer,” “Chemical carcinogenesis - receptor activation,” and “Human cytomegalovirus infection.” Based on the potential targets, we constructed a Glycyrrhiza glabra - active ingredients - target - acute pancreatitis network, revealing six potential active compounds in Glycyrrhiza glabra (Fig. 1E-F).

**Table 1:** Top ten Genes: topological analysis by degree value

Hub Gene	Degree	Eigenvector connectivity	Betweenness centrality	Closeness centrality
MAPK3	70.0	0.25	124.39	0.81
STAT3	66.0	0.25	90.27	0.78
CTNNB1	60.0	0.21	116.70	0.74
SRC	58.0	0.22	82.76	0.73
MAPK1	58.0	0.21	76.74	0.73
RELA	56.0	0.21	77.17	0.72
ESR1	56.0	0.21	76.25	0.72
EGFR	56.0	0.21	57.71	0.72
PRKACA	44.0	0.16	41.87	0.66
CCND1	42.0	0.16	40.38	0.65

### Glycyrrhizin: a promising therapeutic pharmaceutical with high binding affinity to target proteins

**Fig. 2:**
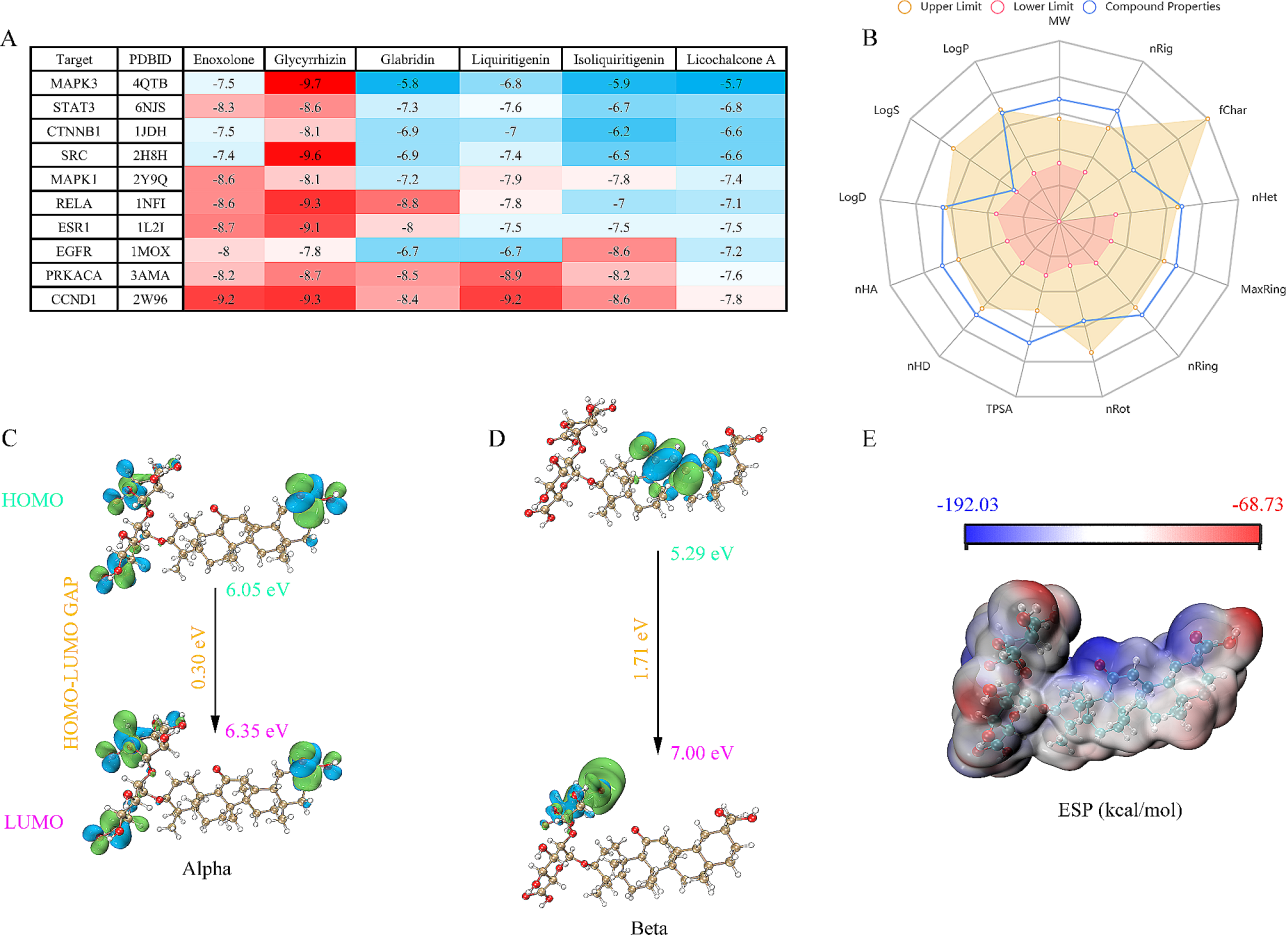
Glycyrrhizin: a promising therapeutic pharmaceutical with high binding affinity to target proteins. **(A)** Heat map illustrating the docking affinity (kcal/mol) between six active components and ten core targets. **(B)** Physicochemical properties of Glycyrrhizin. **(C)** HOMO-LUMO values of alpha orbitals. **(D)** HOMO-LUMO values of beta orbitals. **(E)** MESP map of Glycyrrhizin

Molecular docking analysis was employed to examine the binding patterns between the six main active components and the ten main targets. This computational method is valuable for predicting the interactions and binding affinities of ligands (active components) with their respective protein targets. The target proteins were MAPK3 (PDBID: 4QTB), STAT3 (PDBID: 6NJS), CTNNB1 (PDBID: 1JDH), SRC (PDBID: 2H8H), MAPK1 (PDBID: 2Y9Q), RELA (PDBID: 1NFI), ESR1 (PDBID: 1L2I), EGFR (PDBID: 1MOX), PRKACA (PDBID: 3AMA), and CCND1 (PDBID: 2W96). The affinity values indicate the stability of the receptor-ligand binding, and lower affinity values suggest more stable binding conformations. As shown in Fig. 2A, Glycyrrhizin exhibited strong binding affinity to these 10 target proteins, implying its promising pharmaceutical potential.

To evaluate the physicochemical properties of the candidate compound Glycyrrhizin, we summarized its physicochemical properties, as shown in Fig. 2B. The assessment of drug-likeness properties for candidate drugs is presented in Table 2. Drugs are typically administered orally, mainly absorbed in the intestines, and exert their effects. The volume of distribution (VD) of the candidate compounds ranges from 0.04 to 20 L/kg, indicating good distribution in the body. In the classification of inhibitors, compounds with output values of 0.9 to 1.0 are marked as potential substrates or inhibitors of the corresponding enzymes. Based on the excretion prediction, Glycyrrhizin exhibits moderate phase clearance. In the toxicity evaluation, all compounds showed negative results for the AMES test and skin sensitization, indicating that the compounds are non-mutagenic and non-sensitizing to the skin. The carcinogenicity was also confirmed as negative, suggesting that the compounds do not induce mutations in the biological system. These results indicate that Glycyrrhizin possesses favorable characteristics in terms of intestinal absorption, distribution volume, and toxicity, and exhibits high biological activity, thus holding promising research prospects.

In Fig. 2C-E, the HOMO, LUMO, and MESP structures of glycyrrhizin are presented. The alpha-HOMO-LUMO energy gap is 0.30 eV, and the beta-HOMO-LUMO energy gap is 1.71 eV, with orbital energy levels of 6.05 and 6.35 eV, respectively. The MESP image depicts electron-rich (negative) regions in blue, electron-poor (positive) regions in red, and neutral regions in white. These negative and positive centers play a crucial role in the formation of non-covalent interactions, particularly hydrogen bonds, within the ligand-receptor complex during the molecular docking and MD simulation processes. Understanding the distribution of electrostatic potentials helps to elucidate the nature of interactions between the ligand and receptor, providing valuable insights into the binding mechanisms and stability of the complex.

**Table 2:** ADMET properties of Glycyrrhizin

Abs	Dis	M_et CYP_ (inhibitor)					M_et CYP_ (substrate)					E_x_		T_ox_			
HIA	VD	1A2	2C19	2C9	2D6	3A4	1A2	2C19	2C9	2D6	3A4	CL	*T*_1/2_	AMES	H-HT	SkinSen	Car
+++	0.468	---	---	---	---	---	--	--	--	---	---	0.376	0.116	---	--	---	---

For the classification endpoints, the prediction probability values are categorized into six symbols based on the following intervals: 0-0.1 (---), 0.1–0.3 (--), 0.3–0.5 (-), 0.5–0.7 (+), 0.7–0.9 (++), and 0.9-1.0 (+++)

### Ionic bonds and prolonged stabilization of hydrogen bonding in the binding mode of glycyrrhizin acid to target proteins

**Fig. 3:**
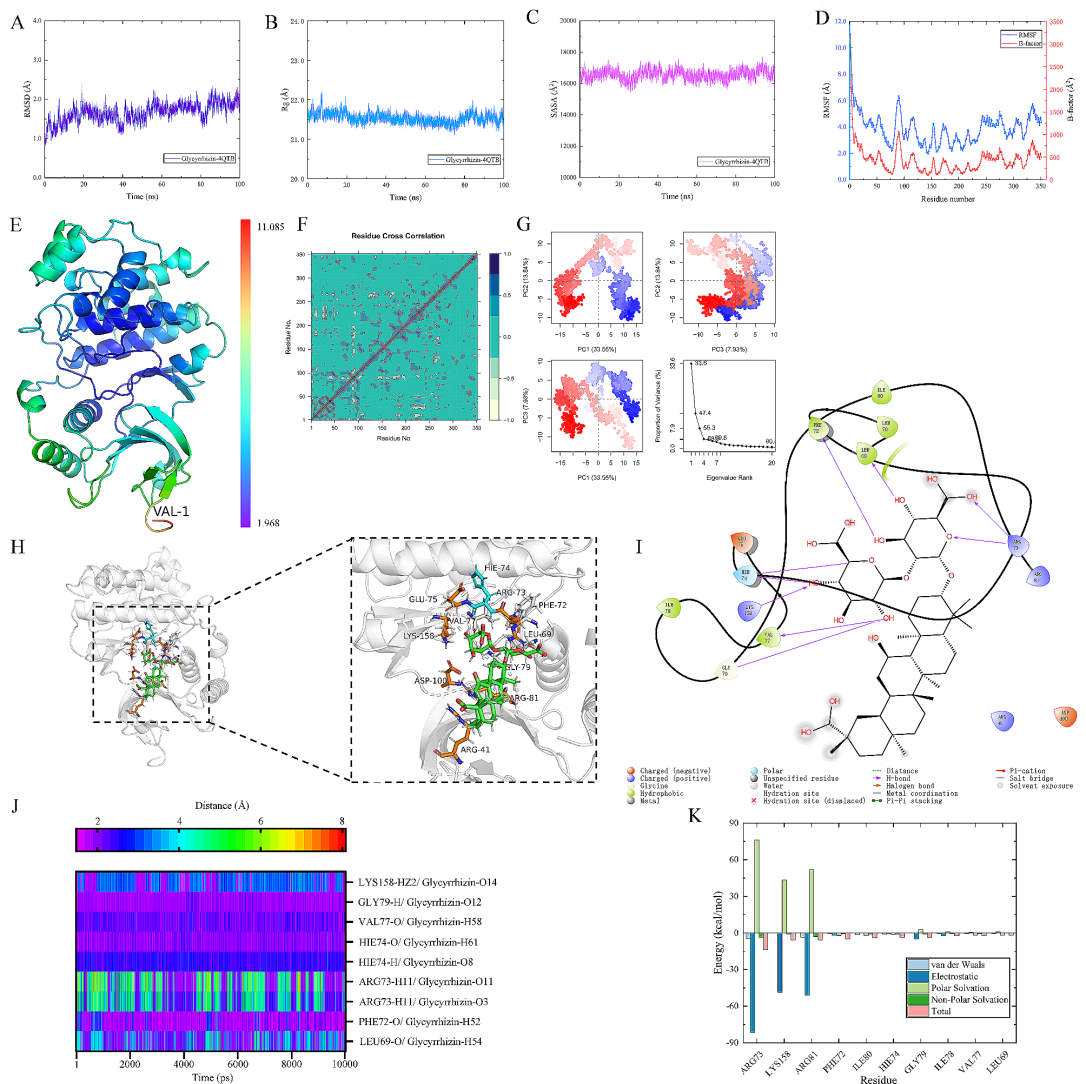
Ionic bonds and prolonged stabilization of hydrogen bonding in the binding mode of Glycyrrhizin acid to target proteins. **(A)** RMSD of the complex during MD simulation. **(B)** Rg of the complex during MD simulation. **(C)** SASA of the complex during MD simulation. **(D)** RMSF and B-factor of the protein. **(E)** Protein secondary structure colored by RMSF values. **(F)** DCCM plot of the complex. **(G)** PCA analysis plot of the complex. **(H)** Interactions within the complex at 100 ns. **(I)** 2D interaction plot of the complex. **(J)** Distances between key residue hydrogen bonds. **(K)** Top 10 energy decomposition residues in the complex interaction

To further unveil the binding mode of Glycyrrhizin, molecular dynamics simulation was conducted. After 100 ns MD simulation of small molecule compound Glycyrrhizin and MAPK3 protein, trajectory analysis was carried out. First, the RMSD of protein and small molecule trajectories was calculated. As shown in Fig. 3A, after 40 ns, the protein-small molecule complex reached a stable state, indicating equilibrium and stability during the simulation. Furthermore, the Rg (Fig. 3B) and SASA (Fig. 3C) analysis supported the observed stability of the protein-small molecule complex at 40 ns. Furthermore, as shown in Fig. 3D-E, the RMSF values of the receptor protein range from 1.968 to 11.085 Å during the 100 ns simulation, indicating the flexibility of the protein and its interactions with the binding site residues, similar to the B-factor observed in crystallography. Interestingly, we observed elevated RMSF values only at the N-terminus of the protein. This indicates increased flexibility and dynamic motion in this region compared to the rest of the protein structure. Such local fluctuations at the N-terminus could potentially play a significant role in protein function and ligand binding. Further investigations are warranted to understand the functional implications of these dynamics and their impact on protein-ligand interactions.

Figure 3F illustrates the conformational variations among snapshots within the simulation trajectory. From the DCCM, it is evident that the protein and the small molecule are tightly bound, and the complex exhibits no significant conformational changes or folding. Figure 3G presents a PCA of the complex protein structures based on the feature scores of the clustered snapshots from the MD simulation trajectory. The color gradient from red to blue indicates the magnitude of conformational changes. Specifically, after Cartesian alignment of the JD C-alpha coordinates, the covariance matrix is computed, and PCA clustering is performed to group protein structures based on their conformational similarities. The first three components account for a total of 55.3% of the variation, showing that there are no major changes or folding in the overall protein conformation. These findings indicate that the MD systems of the complexes are stable and reproducible, rather than being mere artifacts of computational errors.

In Fig. 3H-I, we analyzed the stable conformations obtained from the MD simulations and visualized the interactions using Pymol and Maestro 2021 academic version. In order to assess the hydrogen bond (H-bond) interactions within the complexes, a comprehensive analysis of 10,000 snapshots from the last 10 ns of simulation trajectory was conducted. The results of the examination of hydrogen bond lengths are depicted in Fig. 3J. In Table 3, we analyze the average bond length and occupancy and find long-term stable hydrogen bonds: PHE72-O/ Glycyrrhizin-H52, HIE74-H/ Glycyrrhizin-O8, HIE74-O/ Glycyrrhizin-H61, VAL77-O/ Glycyrrhizin-H58, GLY79-H/ Glycyrrhizin-O12. Our findings highlight the paramount importance of hydrogen bonds in preserving the conformational stability of the complexes. Furthermore, we observed that ionic interactions also play a critical role in maintaining the stability of the protein-ligand complex. This includes key residues, such as ARG41 (negative charge), ARG73 (negative charge), ARG81 (negative charge), LYS158 (negative charge), GLU75 (positive charge), and ASP100 (positive charge). These residues are involved in forming strong ionic bonds with the small molecule ligand, contributing significantly to the overall stability of the complex.

In addition, we employed the MMGBSA method to calculate the binding free energy of the complexes. Figure 3K illustrates the top ten amino acids contributing to the energy decomposition of ΔGbind. The results indicate that ARG73, LYS158, and ARG81 are engaged in robust electrostatic interactions with Glycyrrhizin. In the protein structure, arginine exists in the cationic form, while Glycyrrhizin, as a small molecular compound, possesses negatively charged functional groups. When the cationic form of arginine interacts with the negatively charged functional groups in Glycyrrhizin, an ionic bond is formed, contributing to the stabilization of the protein complex’s structure. This ionic bond plays a crucial role in maintaining the stability and integrity of the protein-Glycyrrhizin complex. This interaction is of significant importance in understanding the structure and function of the protein complex, particularly in drug design and biological research, as it sheds light on the molecular interactions between drugs and proteins and the mechanisms of action within the biological system. This finding implies that the stability of the complex is attributed to multiple interaction forces, particularly ionic bonds, which play a crucial role during the binding process. Moreover, the binding energy and energy decomposition of the complex are illustrated in Table 4.

**Table 3:** The hydrogen bond lengths and occupancy rates

System	Donor-acceptor pair	Average bond length (Å)	Occupancy (%)
Glycyrrhizin-4QTB	LEU69-O/ Glycyrrhizin-H54	2.91 ± 0.91	59.59
	PHE72-O/ Glycyrrhizin-H52	2.08 ± 0.43	94.12
	ARG73-H11/ Glycyrrhizin-O3	3.58 ± 1.12	40.66
	ARG73-H11/ Glycyrrhizin-O11	3.72 ± 1.54	41.09
	HIE74-H/ Glycyrrhizin-O8	2.55 ± 0.24	96.41
	HIE74-O/ Glycyrrhizin-H61	2.08 ± 0.25	99.59
	VAL77-O/ Glycyrrhizin-H58	2.25 ± 0.27	98.77
	GLY79-H/ Glycyrrhizin-O12	2.00 ± 0.17	99.88
	LYS158-HZ2/ Glycyrrhizin-O14	2.92 ± 0.65	34.97

**Table 4:** Binding energy and decomposition energies of the complex system (kcal/mol)

Systems	ΔE_vdw_	ΔE_ele_	ΔG_gas_	ΔE_sol_	ΔG_bind_
Glycyrrhizin-4QTB	-36.91 ± 0.07	82.18 ± 0.59	45.27 ± 0.58	-78.28 ± 0.57	-33.01 ± 0.08

### Glycyrrhizin ameliorates primary pancreatic acinar cell injury by inhibiting MAPK/STAT3/AKT signaling pathway

**Fig. 4:**
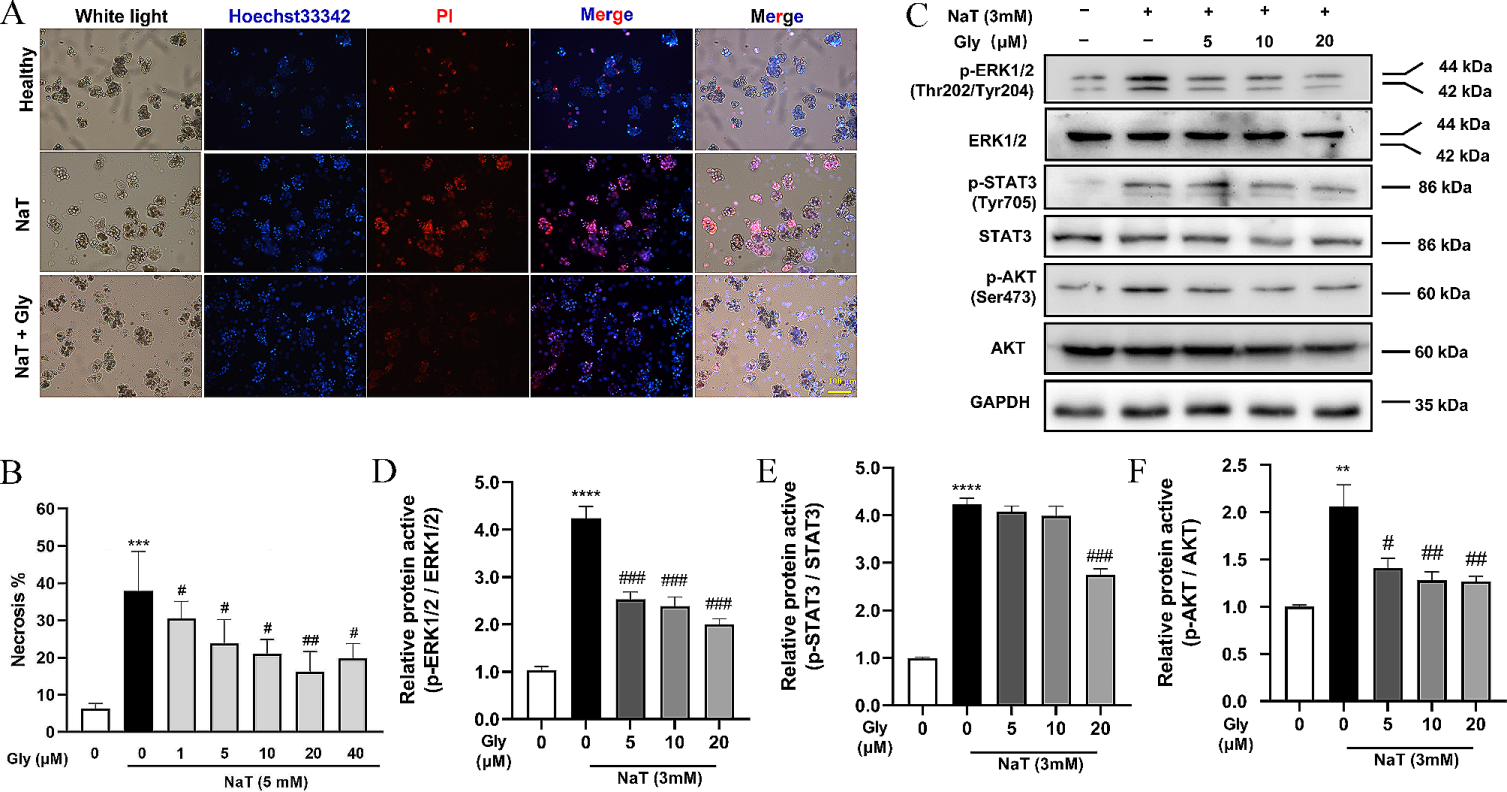
Glycyrrhizin ameliorates primary pancreatic acinar cell injury by inhibiting MAPK (ERK1)/STAT3/AKT signaling pathway. **(A)** Representative images of Hoechst 33,342 and PI staining in primary pancreatic acinar cells. The groups include: Healthy, Model group (NaT), and Treatment group (NaT + Gly). **(B)** Quantitative analysis of sodium taurocholate (NaT)-induced primary pancreatic acinar cell necrosis. The number of PI-stained cells (necrotic) was divided by the number of Hoechst 33,342-positive cells to calculate the percentage of necrosis (%). **(C)** Representative immunoblots for ERK1/2, phospho-ERK1/2 (p-ERK1/2), STAT3, phospho-STAT3 (p-STAT3), AKT, and phospho-AKT (p-AKT) expression in freshly isolated mouse pancreatic acinar cells. Each experiment was performed in triplicate (*n* = 3). **(D-F)** Quantification of protein expression levels from Western Blot analysis. The bar graphs represent the normalized signal intensity ratios for p-ERK1/2 to total ERK1/2, p-STAT3 to total STAT3, and p-AKT to total AKT. Data were quantified and normalized based on appropriate loading controls, and the relative intensities were represented as ratios. *p < 0.05, **p < 0.01, ***p < 0.001 compared to the control group and #*p* < 0.05, ##*p* < 0.01, ###*p* < 0.001, ####*p* < 0.0001 compared to the model group

In order to gain deeper insights into the potential protective effect of Glycyrrhizin on sodium taurocholate (NaT)-induced necrosis in primary pancreatic acinar cells, we performed experiments. NaT stimulation resulted in a notable increase in cell necrosis, as evident by the elevated number of propidium iodide (PI)-positive cells (Fig. 4A). The proportion of necrotic cells rose from 6.24% in the control group to 37.93%. Treatment with glycyrrhizin significantly reduced the proportion of necrotic cells, with the most pronounced inhibitory effect observed at 20 µM glycyrrhizin (Fig. 4B). This data suggests the potential protective role of Glycyrrhizin against NaT-induced necrosis in primary pancreatic acinar cells.

In the context of our study to validate the targeting capability of Glycyrrhizin towards MAPK3 protein (ERK1) and its impact on related signaling pathways, we observed that Glycyrrhizin significantly reduced the levels of phosphorylated proteins ERK1 and STAT3 in pancreatic acinar cells, as well as decreasing phosphorylation of Akt (Fig. 4C-F). This indicates that Glycyrrhizin can inhibit the ERK1 and STAT3 signaling pathways, thereby affecting downstream Akt signaling and improving acinar cell death in acute pancreatitis.

## Discussion

Proteins often rely on interactions with other small molecules in the biological organism to exhibit their biological activities. Therefore, studying the mechanisms of interactions between large protein molecules and small molecules holds significant biological significance in elucidating protein functions [57]. Target identification and determination are primary issues in drug discovery and development. Researchers have developed various target identification techniques, including genomics and proteomics methods, bioinformatics analysis, and using small molecules as probes to explore genome function [58]. The reverse docking technique has also been developed as a target identification method, exploring potential binding proteins for active compounds such as natural products or existing drug molecules [59]. An exemplary case is the protein target of the natural product N-trans-caffeoyltyramine, which was identified using this method and validated through kinase detection and X-ray crystallography [60]. Considering that protein-small molecule interactions in the biological system are dynamic processes, in this study, we performed molecular dynamics simulations on the selected drugs and target proteins. By analyzing various parameters such as Root Mean Square Deviation (RMSD), Root Mean Square Fluctuation (RMSF), Radius of Gyration (Rg), and Solvent Accessible Surface Area (SASA), we obtained a highly detailed depiction of the molecular motions. Principal Component Analysis (PCA) was utilized to reduce the dimensionality of the covariance matrix of all conformations with respect to the average structure, enabling the extraction of relevant fluctuations from the MD simulation trajectories. By integrating these various analyses, we gain a comprehensive understanding of the dynamic behavior of the drug-protein complex. This detailed molecular motion image enhances our knowledge of the binding process and lays the foundation for further structure-based drug design and optimization. Overall, molecular dynamics simulations serve as a valuable tool in the exploration of protein-drug interactions and contribute to the rational design of effective therapeutic agents [61]. The integration of computational approaches with experimental techniques continues to drive progress in target identification, enhancing the efficiency and success rate of drug discovery efforts. As our understanding of protein-small molecule interactions deepens, it paves the way for the development of novel therapeutic agents and the exploration of uncharted areas in biomedical research.

AP is a severe inflammation of the pancreas characterized by abdominal pain and elevated pancreatic enzyme levels in the blood. Initial injury leading to AP causes acinar cell death and inflammatory responses [62]. AP is characterized by complex pathological changes, and its pathogenesis is not fully understood. However, substantial evidence supports that acinar cell injury is the initiating and central event in AP. Various factors lead to a series of intracellular events, including calcium overload, oxidative stress, mitochondrial energy metabolism disorders, endoplasmic reticulum stress, premature activation of pancreatic enzymes, activation of nuclear transcription factors, programmed cell death, and damage-associated molecular pattern molecules, activating related inflammatory pathways, leading to systemic inflammatory response syndrome and multiple organ failure [63]. In recent years, numerous clinical studies have shown that integrative Chinese and Western medicine treatment significantly improves AP efficacy. Chinese herbal formulae and monotherapy for AP are increasingly recognized as effective and safe, with mounting evidence suggesting that Chinese medicine can reduce serum and urinary amylase levels, decrease capillary permeability, inhibit the production of inflammatory cytokines, suppress neutrophil activation, and alleviate pancreatic injury [7].

Liquorice is a perennial herb commonly used in traditional Chinese medicine, and it is essential to clarify its bioactive chemical components and mechanisms of action [64]. Network pharmacology is a commonly used research method for identifying active compounds and targets, with advantages of comprehensiveness, systematicity, and integrality, which suits the characteristics of Chinese medicine with multiple compounds, targets, and pathways [65]. In this study, we established a network pharmacology approach integrating target identification, network analysis, and KEGG pathway analysis, combined with molecular docking, molecular dynamics simulations, density functional theory calculations, ADMET prediction, and other multiple analyses, as well as in vitro studies, to elucidate the potential molecular mechanisms of licorice in treating AP. Our research found that Glycyrrhiza glabra exhibited multi-target and multi-pathway characteristics in treating AP. After molecular docking and molecular dynamics simulations, we discovered that Glycyrrhizin could bind to multiple target proteins with good selectivity. In the binding with MAPK3 protein, ionic bonds and hydrogen bonds significantly contributed to conformational stability, especially for the key residues ARG73, LYS158, and ARG81. The overall binding free energy of the complex reached − 33.01 ± 0.08 kcal/mol, indicating that MAPK3 might be a potential target during the treatment of AP by Glycyrrhizin.

Studies have demonstrated that glycyrrhizic is capable of directly binding to lipoxygenase, leading to the generation of inflammatory mediators [7]. Additionally, glycyrrhizic selectively inhibits the phosphorylation triggering of these inflammatory mediators, which are primarily enzymes [66]. Glycyrrhizic and its derivatives exert specific inhibition on the production of crucial inflammatory chemokines, such as IL-8 and eotaxin 1, which play pivotal roles in leukocyte chemotaxis during inflammation. Moreover, glycyrrhizic and its derivatives effectively neutralize the release of these pro-inflammatory chemokines [67]. This finding highlights their potential as promising candidates for anti-inflammatory therapeutics. In addition, in alcoholic hepatitis rat models, glycyrrhizin exhibits inhibitory effects on the secretion of human growth-regulated oncogene/keratinocyte chemoattractant (GRO/KC), granulocyte-macrophage colony-stimulating factor (GM-CSF), vascular endothelial growth factor (VEGF), and intercellular adhesion molecule 1 (ICAM-1). Additionally, glycyrrhizin also suppresses GM-CSF levels [68]. Glycyrrhizin also inhibits phospholipase A2/arachidonic acid (PLA2/ARA) pathway metabolites, such as prostaglandin-E2 (PGE2) or prostacyclin 2, thromboxane 2 (TXA2), and leukotriene B4 (LTB4) [69]. These findings suggest that the anti-inflammatory effects of glycyrrhizic and glycyrhizin are attributed to their direct binding to cellular membrane components, such as lipocortin I (LC-1), or enzymes like PLA2. Notably, glycyrrhizin significantly reduces the concentrations of matrix metalloproteinase-9 (MMP-9) and intercellular adhesion molecule-1 (ICAM-1). Moreover, it enhances the activities of glutathione peroxidase (GSH-Px) and superoxide dismutase (SOD), along with the secretion of phosphorylated Akt (p-Akt) and phosphorylated extracellular signal-regulated kinase (p-ERK) [70]. Moreover, Glycyrrhizin inhibits the stimulation of signal transducers and activators of transcription-3 (STAT3), decreases the upregulation of ICAM-1 and P-selectin secretion, reduces the configuration of polyadenosine diphosphate-ribose (pADR) and nitrotyrosine (NTS), and decreases polymorphonuclear neutrophil infiltration (PMN) [71]. In addition, clinical research has highlighted the central role of Monocyte Chemoattractant Protein-1 (MCP-1) in the development of AP. The heightened MCP-1 levels observed in AP patients sharply contrast with the remarkable capacity of Glycyrrhizin to significantly reduce serum MCP-1 levels in experimental mouse models of AP [72]. Yaser Panahi’s previous research demonstrated that glycyrrhizin directly downregulates the levels of CCL2 and CXCL2 in acinar cells stimulated by cerulein, thereby improving acinar cell damage [73]. The work of Yam Nath Paudel has illuminated the pharmacological potential of Glycyrrhizin by suppressing the activity of High Mobility Group Box 1 (HMGB1), leading to the diminution of inflammatory cytokine levels, release, and expression of HMGB1 [74]. This phenomenon is further corroborated by the investigations of Pan YL and Yildirim AO, providing substantiation that Glycyrrhizin could effectively alleviate AP in murine models through its potential to suppress serum inflammatory mediators and decrease HMGB1 expression within pancreatic tissue [75, 76]. Notably, the administration of Glycyrrhizin has demonstrated the ability to mitigate AP in mice, while concurrently mitigating the infiltration of pancreatic tissue, particularly neutrophil infiltration. Ke Xiang et al. observed that glycyrrhizin enhances the survival rate and ameliorates pancreatic injury in traumatic pancreatitis by suppressing the expression of pro-inflammatory cytokines, including HMGB1 [22]. However, the exact molecular mechanisms that underlie the therapeutic efficacy of Glycyrrhizin in AP treatment require further elucidation. As a result, our research employs computational biological screening to identify potential therapeutic targets for Glycyrrhizin in AP treatment, while simultaneously investigating its influence on the critical interplay of acinar cell injury and the associated molecular pathways. In our study, we found that Glycyrrhizin can ameliorate pancreatic acinar cell necrosis through in vitro experiments. Western blot experiments revealed that this effect was achieved by inhibiting the phosphorylation activation of ERK1/2 and STAT3 proteins, as well as downstream AKT protein. These findings confirm that Glycyrrhizin, as a significant active component of Glycyrrhiza glabra, can target the MAPK3 protein, improving pancreatic acinar cell necrosis and thus providing therapeutic value for acute pancreatitis.

## Conclusion

In conclusion, our study utilized a combination of network pharmacology, molecular docking, and MD simulations to elucidate the role of Glycyrrhizin as a critical bioactive component within Glycyrrhiza glabra. We have identified its ability to form a stable complex with the MAPK3 protein through the involvement of ionic bonds and hydrogen bonds. Furthermore, our in vitro experiments have revealed that Glycyrrhizin exerts inhibitory effects on the ERK1/2, STAT3, and downstream p-AKT signaling pathways, leading to a remarkable amelioration of acinar cell death in acute pancreatitis. The comprehensive approach adopted in this research has laid the groundwork for an in-depth exploration of traditional Chinese medicine ingredients, their associated targets, and underlying mechanisms of action in the context of AP treatment. Such findings could have significant implications for future drug development and clinical applications.

### Electronic supplementary material

Below is the link to the electronic supplementary material.

Supplementary Material 1

## Data Availability

The data that support the findings of this study are available from the corresponding authors upon reasonable request. Some data may not be made available due to privacy or ethical restrictions.
