# Supplementary material for: Network pharmacology and in vitro experimental verification unveil glycyrrhizin from glycyrrhiza glabra alleviates acute pancreatitis via modulation of MAPK and STAT3 signaling pathways
Source: BMC Complement Med Ther. 2024 Jan 27;24:58. doi: 10.1186/s12906-024-04372-x (PMC10821312; doi:10.1186/s12906-024-04372-x)
Supplement: Supplementary file 1 — Supplementary Material 1 [file 12906_2024_4372_MOESM1_ESM.docx]

**SUPPLEMENTARY INFORMATION for:**

**Network pharmacology and *in vitro* experimental verification unveil Glycyrrhizin from Glycyrrhiza glabra alleviates acute pancreatitis via modulation of MAPK and STAT3 signaling pathways**

Zhang et al

**The western blotting bands in the study**

**Western blots for figure 4**
